# Coregistered optical coherence tomography and frequency-encoded multispectral imaging for spectrally sparse color imaging

**DOI:** 10.1117/1.JBO.25.3.032008

**Published:** 2019-11-21

**Authors:** Xavier Attendu, Marie-Hélène Bourget, Martin Poinsinet de Sivry-Houle, Caroline Boudoux

**Affiliations:** a Polytechnique Montréal, Centre d’Optique Photonique et Lasers, Department of Engineering Physics, Montréal, Canada; b Castor Optics Inc., St-Laurent, Canada

**Keywords:** optical coherence tomography, multispectral imaging, color reconstruction, frequency domain multiplexing, double-clad fiber coupler

## Abstract

We present a system combining optical coherence tomography (OCT) and multispectral imaging (MSI) for coregistered structural imaging and surface color imaging. We first describe and numerically validate an optimization model to guide the selection of the MSI wavelengths and their relative intensities. We then demonstrate the integration of this model into an all-fiber bench-top system. We implement frequency-domain multiplexing for the MSI to enable concurrent acquisition of both OCT and MSI at OCT acquisition rates. Such a system could be implemented in endoscopic practices to provide multimodal, high-resolution imaging of deep organ structures that are currently inaccessible to standard video endoscopes.

## 1 Introduction

Optical coherence tomography (OCT) is a powerful imaging modality that relies on interferometry to image subsurface structures.^1^ It provides cross-sectional images of tissue morphology up to several millimeters deep, with axial resolutions ranging from 1 to 20  μm.^2^^,^^3^ Using optical fiber technology, OCT has been adapted to endoscopic imaging in various fields, which include cardiology,^4^ gastroenterology,^5^^–^^7^ laryngology,^8^ urology,^9^ and gynecology.^10^ Furthermore, highly miniaturized OCT probes have enabled access to deep organ structures that are inaccessible to standard video endoscopes.^11^ As such, OCT has the potential to expand current diagnostic capabilities in endoscopic practices.

Despite its great promises, OCT suffers from certain shortcomings. First, OCT produces images that differ significantly from the color images obtained in standard white light video endoscopy (WLE), the standard of care for initial evaluation in many instances in gastrointestinal and pulmonary endoscopy.^12^^,^^13^ Differences such as the imaging scale, the perspective (cross-sectional versus *en-face*), and the lack of color may potentially add to the challenges of interpretation and clinical translation for OCT. Second, in its standard implementation, OCT provides only limited information regarding the molecular composition of the sample. While some variants of OCT with enhanced molecular sensitivity exist, they typically present some performance trade-offs or limitations with respect to speed, sensitivity, or experimental complexity.^11^^,^^14^ An alternative to enhancing the molecular sensitivity of OCT is the multimodal approach, whereby OCT is combined with other complementary techniques. For instance, OCT has previously been combined with fluorescence imaging,^15^^,^^16^ multiphoton microsocopy,^17^ Raman spectroscopy,^18^ fluorescence lifetime imaging,^19^^,^^20^ and hyperspectral imaging (HSI).^21^ In each of these instances, the added modality provides molecular insight, complementary to the morphological imaging with OCT.

Another candidate for combination with OCT is multispectral reflectance imaging (MSI). This technique is similar to HSI, where images of a scene are captured at multiple spectral bands.^22^ The difference between the two modalities lies in the number of spectral bands captured, where MSI has a few and HSI has many. In both cases, this results in a three-dimensional (3-D) dataset with two spatial dimensions and one spectral dimension. Each reflectance spectrum carries information on the attenuation properties of the studied sample, which can be used to infer molecular and/or physiological properties. While HSI may be better suited to detect subtle spectral differences, it also generates a significant amount of redundant data as well as additional experimental and computational complexity. In the case of MSI, a careful selection of the spectral bands allows the simplification of the setup without compromising molecular imaging capabilities, albeit for a selected set of molecular markers. Furthermore, the spectral reflectance information in the visible (VIS) range can be used to reconstruct the color of the sample. This can provide clinicians with a familiar perspective as well enable the continued use of current WLE evaluation protocols, where color is key in the identification of physiological features.^23^^,^^24^

To this effect, we propose to combine OCT with a multispectral reflectance imaging system optimized to reproduce the sample’s color as it would appear under white light endoscopy. Both modalities are combined in a single optical fiber through the use of a double-clad fiber coupler (DCFC).^25^ This allows for simultaneous OCT and MSI while conserving the potential for miniaturization. The miniaturization of the proposed system into a small diameter endoscopic probe could enable concurrent OCT, color imaging, and select spectral analyses of deep organ structures that are not accessible to standard WLE endoscopes. Both modalities are acquired concurrently and are intrinsically coregistered. We first present an optimization protocol for the selection of the MSI wavelengths and their relative intensities. We then demonstrate its implementation in a bench-top system where all MSI spectral bands are multiplexed in the frequency domain. Finally, we evaluate the system’s performance for reflectance measurements, color reconstruction, and high-resolution imaging.

## 2 Theory

### 2.1 Fundamentals of Color Reconstruction

The color appearance of an object can be reconstructed based on the spectral power distribution of the light reflected off the object that is then detected (using the eye or a camera). This reflected spectrum is defined as the multiplication of the illumination spectrum, I(λ), and the spectral reflectance of the object, R(λ). Using the standardized CIE 1931 color-matching functions $\overset{¯}{x}(\lambda )$, $\overset{¯}{y}(\lambda )$, and $\overset{¯}{z}(\lambda )$ (2° Standard Observer), this spectrum may be converted into a set of color coordinates in CIE XYZ color space.^26^ The mathematical operations presented in Eqs. (1)–(4) can, therefore, be used to simulate the color appearance of a sample of known reflectance under any arbitrary illumination:

$$X=\frac{1}{N}\int_{\lambda }I(\lambda )R(\lambda )\overset{¯}{x}(\lambda )d\lambda ,$$ (1)

$$Y=\frac{1}{N}\int_{\lambda }I(\lambda )R(\lambda )\overset{¯}{y}(\lambda )d\lambda ,$$ (2)

$$Z=\frac{1}{N}\int_{\lambda }I(\lambda )R(\lambda )\overset{¯}{z}(\lambda )d\lambda ,$$ (3)

$$N=\int_{\lambda }I(\lambda )\overset{¯}{y}(\lambda )d\lambda ,$$ (4)

where X, Y, and Z represent the color coordinates and N is a normalization factor. In Eqs. (1)–(4), the integration range covers the VIS spectral range, i.e., λ=[380,780]  nm. The color coordinates in the XYZ color space can then be transformed into RGB coordinates for display or into other color spaces for further analyses.^26^^,^^27^

### 2.2 Optimizing Spectrally Sparse Color Reconstruction

Equations (1)–(4) show that the XYZ coordinates, and therefore the color, of a sample differ when illuminated with a broadband (white) light source as opposed to several narrow-linewidth lasers. However, it is possible to select the number, the wavelengths, and the relative intensities of such lasers in order for the color appearance to be as close as possible to that under a reference illuminant. In the case of such multilaser scheme, the illumination spectrum, I(λ), can be approximated as a sum of delta functions multiplied by relative intensity terms, as

$$I(\lambda )=\overset{n}{\underset{i}{\sum }}I(\lambda_{i})\delta (\lambda_{i}),$$ (5)

where the subscripts i and corresponding $\lambda_{i}$ refer to the different laser lines used. This approximation is valid under the assumption that the reflectance spectrum and the color-matching function are almost constant over the spectral width of the laser line. By inserting Eq. (5) into Eqs. (1)–(4), the color coordinates can be expressed as sums rather than integrals:

$$CC=\frac{1}{N}\overset{n}{\underset{i}{\sum }}I(\lambda_{i})R(\lambda_{i})f_{\mathrm{cm}}(\lambda_{i}),$$ (6)

$$N=\overset{n}{\underset{i}{\sum }}I(\lambda_{i})\overset{¯}{y}(\lambda_{i}),$$ (7)

where CC are the color coordinates (X, Y, and Z) and $f_{\mathrm{cm}}(\lambda )$ are the corresponding color-matching functions. In Eq. (6), the constant factor that relates the integral of $I(\lambda )R(\lambda )f_{\mathrm{cm}}(\lambda )$ over the narrow spectral width of a single laser line to the multiplication of single terms $I(\lambda_{i})R(\lambda_{i})f_{\mathrm{cm}}(\lambda_{i})$ will be factored out by the normalization by N.

Using Eqs. (1)–(4) for broadband illumination or Eqs. (5)–(7) for multilaser illumination, it is possible to compute the color coordinates under any illumination for a sample of known reflectance. We can then optimize our choice of wavelengths and relative intensities to minimize the color difference between the color obtained under broadband illumination and the one obtained under multilaser illumination. The color difference, typically denoted as ΔE, can be quantitatively assessed by comparing two sets of color coordinates. Associated with the characterization of color difference is the concept of just noticeable difference (JND). In color vision, the JND refers to the minimum ΔE that can be detected at least half of the time by a human observer.^28^ In other words, it represents the limit of human perception. By definition, the JND is set to ΔE=1.^26^^,^^29^ A common measure of color difference is obtained by computing the Euclidean norm of the difference between two color coordinate vectors in CIELAB, a color space derived from CIE XYZ.^30^ However, this color difference metric is known to present perceptual nonuniformities meaning that a given ΔE is more noticeable for certain colors than for others.^26^^,^^27^ This may then lead to imbalanced optimization, favoring certain colors over others. We, therefore, selected the $\Delta E_{2000}$ color difference metric, as it was specifically developed to have improved perceptual uniformity.^31^^,^^32^

Mathematically, the optimization problem presented above can be expressed as

$$\underset{\lambda_{i},I(\lambda_{i})}{\text{minimize}}\;\frac{1}{2}{\left\|\Delta E[\lambda_{i},I(\lambda_{i})]\right\|}^{2};\;\text{subject to }\begin{array}{l} \lambda_{i}\in [380,780]\mathrm{nm};\\ I(\lambda_{i})\geq 0 \end{array}.$$ (8)

In this system, $\Delta E[\lambda_{i},I(\lambda_{i})]$ is a vector containing the $\Delta E_{2000}$ color differences computed for multiple samples, $\lambda_{i}$ are the n selected wavelengths and $I(\lambda_{i})$ are the relative intensities associated with each wavelength. To achieve optimal reconstruction over a broad range of colors, it is not sufficient to perform this optimization on a single sample. Indeed, this may lead to a wavelength/intensity configuration that performs well for the selected sample but very poorly for samples with different reflectance spectra and colors. Therefore, the process must be performed for multiple different output colors simultaneously, in order to optimize the overall color rendering ability of the multiwavelength illumination.

It is interesting to note that this optimization can also be performed with fixed wavelengths. Such an optimization yields the relative intensities that result in optimal color reconstruction for the selected spectral lines. This scenario is useful as it is more representative of reality, where lasers are not available at any arbitrary wavelength or if nonoptimal wavelengths are used (as is the case for the experimental system presented in this work) due to practical limitations such as cost, availability, or other technical limitations. As such, the optimization over all wavelengths and intensities can be used to identify the optimum wavelengths ranges. The intensity-only optimization can then be performed using commercially available wavelengths that closely match the optimal ones, in order to realistically evaluate color rendering capabilities.

## 3 Materials and Methods

### 3.1 Numerical Optimization for Color Reconstruction

The color-rendering performance of a multiwavelength laser source was simulated and optimized against that of a xenon white arc lamp (SLS401, Thorlabs, Newton, USA), as xenon illumination is commonly used in upper gastrointestinal WLE. However, the same analysis can be performed for other reference illuminants. The 24 reflectance spectra of a Macbeth ColorChecker, also known as a ColorChecker Classic (X-rite, Grand Rapids, USA), were used as the colored samples in the optimization process.^33^ The color coordinates of each square under the reference illumination were computed using the corresponding reflectance spectra and Eqs. (1)–(4) (reflectance data available online^33^). The optimization problem described in Eq. (8) was solved using the *fmincon* solver from MATLAB (MathWorks, Natick, USA) to identify the n optimal wavelengths and their relative intensities. The optimization was carried out for three to five wavelengths, using the $\Delta E_{2000}$ color difference in the objective function [Eq. (8)]. The initial point for the optimization was set to equally spaced wavelengths between 450 and 650 nm with equal initial intensity. We also performed several intensity-only simulations using commercially available wavelenghts that closely matched the optimal wavelengths identified through the optimization process.

To validate the accuracy of color reconstruction for biological samples, we simulated the appearance of the inner mucosa of a lip under multispectral illumination. For this purpose, we used a reflectance hyper-cube (i.e., an image with a full reflectance spectrum for each pixel) acquired using a hyperspectral camera (Specim IQ, Specim Spectral Imaging Ltd, Oulu, Finland). During the acquisition of hyperspectral images, a white reference reflector (SRS-99, Labsphere, North Sutton, USA) was included in the field of view (FOV) from which we determined the reflectance spectra.

### 3.2 System Description

**Fig. 1:**
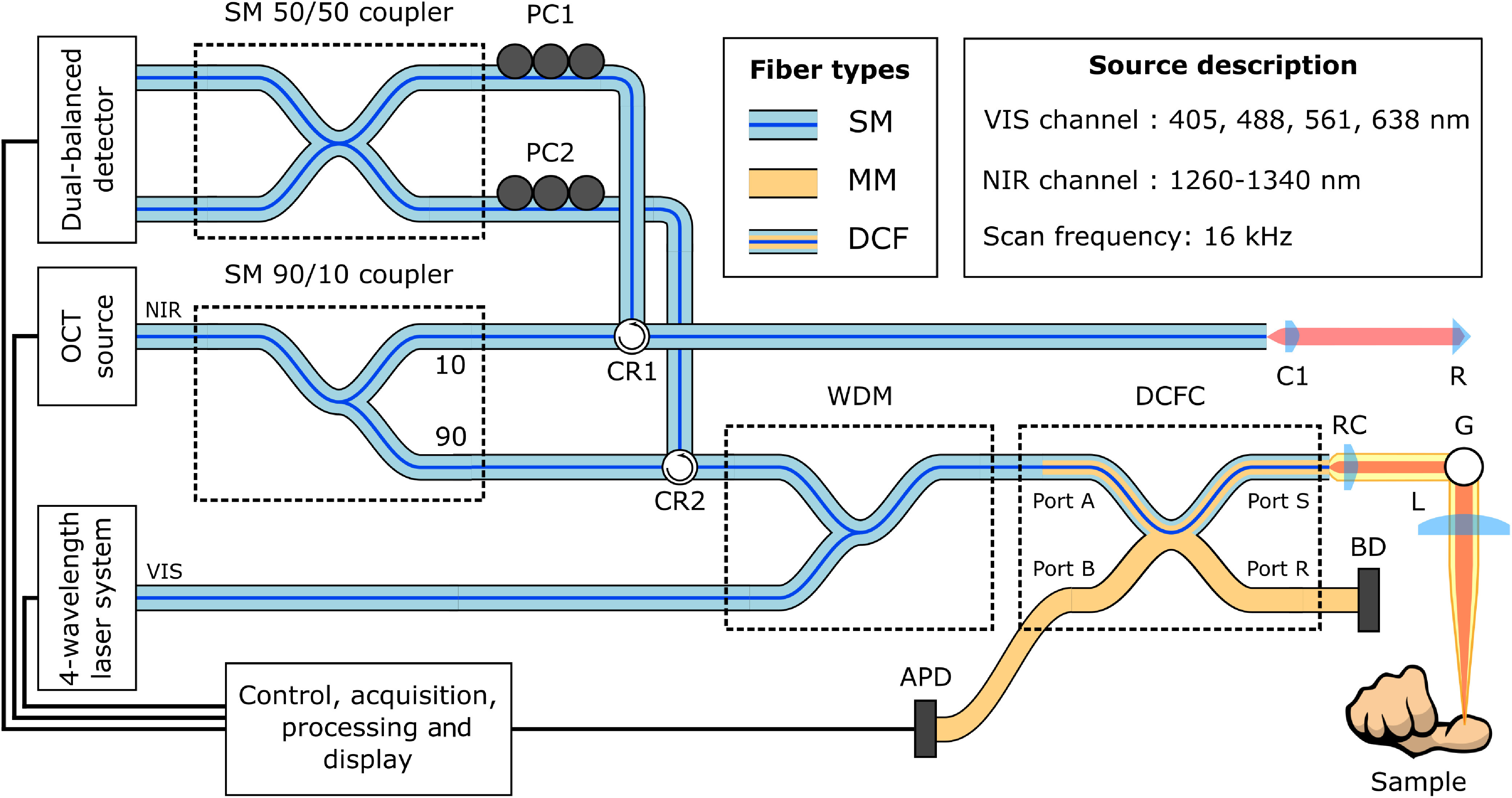
Experimental setup. CR1, CR2, fiber circulators; WDM, wavelength-division multiplexer; DCFC, double-clad fiber coupler; SM, MM, DCF, single mode, multimode, and double-clad fiber, respectively; C1, collimator; RC, reflective collimator; BD, beam dump; G, 2-D galvanometer scanning system; L, focusing lens; R, mirror/retroreflector; PC1, PC2, paddle polarization controllers; APD, avalanche photodetector.

The experimental setup is shown in Fig. 1. In this setup, we use two distinct light sources. The first is a wavelength-swept laser scanning the range from 1260 to 1340 nm for OCT (OCS1300SS, Thorlabs, Newton, USA) with a sweep frequency of 16 kHz. The second source is a four-wavelength system that combines the output of four separate lasers (405, 488, 561, and 638 nm) into a single fiber (L4Cc, Oxxius, Lannion, France) for MSI. These wavelengths were not selected through the optimization process detailed above but came preconfigured with the multilaser system. In this multiwavelength source, each laser can be controlled and modulated independently. The output of the OCT source is first split into a sample and reference arm using a 90/10 single-mode fiber coupler (TW1300R2A2, Thorlabs). The illumination signals of both modalities are then combined into a single SMF28 fiber using a home-built wavelength-division multiplexer (WDM). The output of the WDM is then spliced to a DCFC (DC1300LEFA, Castor Optics, inc., St-Laurent, Canada). The illumination light is transported in the core of the double-clad fiber (DCF) from port A to port S, where it is collimated using a reflective collimator (RC, RC04APC-P01, Thorlabs). The collimated beam is scanned using a two-axis galvanometer system (G, GVS002, Thorlabs) and a VIS-coated achromatic doublet lens (L, AC254-075-A, Thorlabs) in a telecentric configuration. Light backscattered from the sample is then collected in both the DCF core and inner cladding. Light in the inner cladding is extracted from port S to port B by the DCFC, where it is measured by a fast avalanche photodetector (APD, APD120A, Thorlabs). As the spectral range of the photodetector is from 400 to 1000 nm, it is not sensitive to OCT wavelengths and, therefore, only detects the MSI signal. Moreover, the frequency filtering used to extract the MSI signal (see Sec. 3.3) further attenuates any potential noise from the OCT signal. Light collected into the fiber core is transported back to the WDM. As this component is reciprocal, VIS light is mostly extracted back to the VIS laser. In this configuration, it is not necessary to include any isolation to protect the laser source, as the intensity levels returning to the laser are minimal. The returning OCT signal is transported, via a fiber circulator (CR2, CIR-1310-50-APC, Thorlabs) to a 50/50 single-mode fiber coupler (TW1300R5A2, Thorlabs) where it interferes with the signal from the reference arm. The reference arm consists of a segment of SMF28 fiber length-matched to the total fiber length of the WDM and DCFC, a collimator (C1, CFS5-1310-APC, Thorlabs), a free-space segment matching the space between the fiber tip at port S and the sample and a mirror (R). It is important to note that the optical properties of the DCF used in the DCFC are matched to that of SMF28 fiber. As such, the component does not induce dispersion in the OCT signal and the fiber lengths in the reference arm can be matched one-to-one with those in the sample arm. Paddle polarization controllers (PC1 and PC2, FPC560, Thorlabs) are used in both interferometer arms to optimize the interference signal. The interference signal after the 50/50 coupler is detected using a balanced detector (DB Detection, PDB440C, Thorlabs). The OCT signal is unaffected by any residual VIS light as it lies outside of the spectral range of the DB detector. A custom A-line trigger (not shown) is used to trigger both OCT A-line and MSI acquisition. Custom LabView (National Instruments, Austin, USA) software is used to control the scan and synchronized acquisition of both modalities. The acquisition is performed using an AlazarTech ADC (ATS9440, AlazarTech, Pointe-Claire, Canada) with 20  MS/s sampling frequency. Scanning is synchronized and driven using a National Instruments DAC (PCI-6111, National Instruments).

### 3.3 MSI Signal Acquisition and Processing

All four spectral components of the MSI are acquired simultaneously and at the same pixel rate as the OCT A-line rate (16 kHz) through the use of frequency-domain multiplexing. Each laser in the four wavelength source is modulated sinusoidally at a distinct frequency: 700, 800, 900, and 1000 kHz for 405, 488, 561, and 638 nm, respectively. Using Fourier analysis, the collected intensity for each wavelength is identified and used to compute the reflectance as follows:

$$R(f_{i})=\frac{I_{\mathrm{sam}}(f_{i})-I_{\text{dark}}(f_{i})}{I_{\mathrm{ref}}(f_{i})-I_{\text{dark}}(f_{i})},$$ (9)

where each intensity measurement $I(f_{i})$ corresponds to the height of the peak in the Fourier domain at the corresponding frequency, $f_{i}$. These values can be measured by performing a fast Fourier transform (FFT) on the signal after DC removal. A Hanning window is also applied prior to FFT to remove potential side lobes due to signal truncation. The dark measurement is performed without a sample, and the reference measurement is performed using a 99% white diffuse reflectance standard (SRS-99, Labsphere, North Sutton, USA). It is important to note that the reflectance measurements do not require the illumination intensities to match those determined in the optimization procedure. Indeed, once the reflectance is known at the selected wavelengths, the color appearance can be simulated in postprocessing. During the reference measurements, the axial position of the reference sample is monitored with OCT and always positioned at the same location to avoid any variations in the illumination and collection areas. The axial position of the first surface of samples is likewise controlled.

Selecting the modulation frequencies for the multilaser source requires considering certain properties of the FFT. For the MSI acquisition to run in parallel with the OCT acquisition, the longest sampling time corresponds to that of a single A-line. As such, the smallest resolvable frequency difference is given by $\Delta f_{\min}=1/\tau_{A-\text{line}}=f_{A-\text{line}}$. The MSI modulation frequencies must, therefore, be selected such that they are sufficiently far apart to be resolvable. The shape of the modulation must also be considered. Ideally, analog modulation with a sinusoidal waveform should be used as it results in a single peak in the Fourier domain. However, most commercial lasers only permit analog modulation up to several MHz, which might be restrictive for higher acquisition speeds. Digital (TTL) modulation typically allows much higher modulation frequencies but with a square waveform. This results in multiple peaks in the Fourier domain at multiples of the fundamental modulation frequency. To avoid cross-talk between the different wavelengths, care should be taken that the fundamental frequency peaks do not overlap with secondary peaks from another laser. It is important to point out that not all lasers allow high-speed modulation. In particular, diode-pumped solid-state lasers (typical wavelength range from 530 to 600 nm) do not usually permit such high modulation frequencies. It is then necessary to combine these lasers with an external modulator. In our experimental setup, the 561-nm channel of the multilaser system included an acousto-optic modulator with a 3-MHz bandwidth.

All of these criteria were respected when defining our acquisition parameters. All frequencies and their spacing are greater than $f_{\min}=16\;\mathrm{kHz}$, below the 3 MHz analog modulation limit, and below the maximum measurable frequency of 10 MHz defined by the 20-MS/s sampling rate.

### 3.4 OCT Signal Acquisition and Processing

The OCT signal is sampled simultaneously with the MSI signal, using the same multichannel ADC. Sampling is carried out at 20  MS/s with 1312 samples per A-line. The k-clock output of the OCT swept-laser is not used as one of the two signals would inevitably have to be resampled. The OCT signal is first corrected for background and high-pass filtered to remove low-frequency components. It is then resampled using a cubic spline to achieve k-linear sampling and multiplied by a complex window for dispersion compensation. The resampling indices and the appropriate dispersion correction vector were identified using a calibration method based on two mirror measurements.^34^ Dispersion compensation is particularly important in this scenario as the use of the WDM induces a significant dispersion mismatch between the two interferometer arms.

## 4 Results

### 4.1 Numerical Optimization for Color Reconstruction

**Fig. 2:**
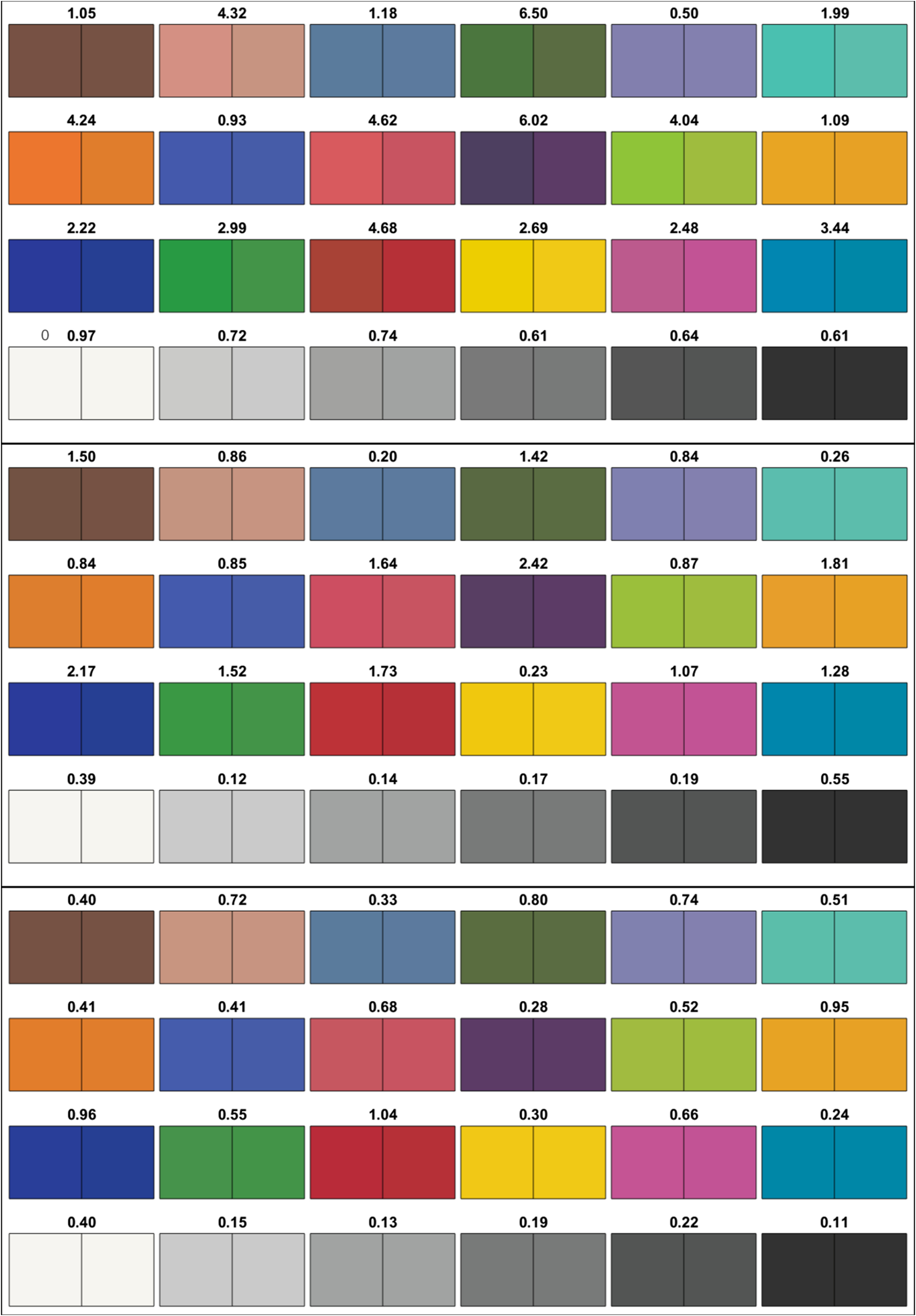
Reconstructed color appearance of the ColorChecker under the reference illuminant (right square) and multispectral illumination schemes presented in Table 1 (left square: from top to bottom 3, 4, and 5λ, respectively). The $\Delta E_{2000}$ color difference is indicated above each sample.

**Fig. 3:**
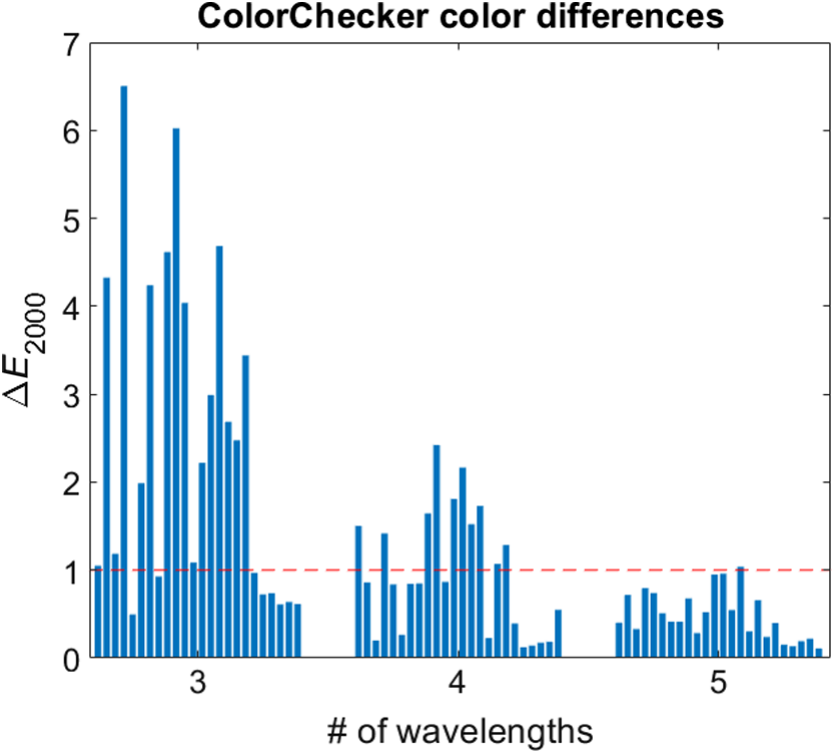
Color difference values for the different wavelength configurations listed in Table 1. Each bar represents one of the 24 colored squares (ordered from left to right and top to bottom in Fig. 2). The horizontal dashed line indicates the JND.

The results of the optimization protocol with three to five illumination wavelengths are presented in Table 1. All relative intensities are normalized to the intensity of the first wavelength. In this table, the $\left\langle \Delta E_{2000}\right\rangle$ column refers to the mean color difference across the 24 colored squares of the ColorChecker and ${\left\langle \Delta E_{2000}\right\rangle }_{\text{lip}}$ column to the mean color difference over all image pixels of the lip mucosa. Table 1 shows that color reconstruction below the JND threshold can be achieved with as few as four wavelengths for the ColorChecker. Figure 2 shows the color appearance of the ColorChecker under the reference illumination (xenon) and under the multispectral illumination configurations described in Table 1. It should be noted that the color appearance of Fig. 2 may vary slightly depending on the color calibration of the monitor or the printer used by the reader. The $\Delta E_{2000}$ values for all 24 color squares of the ColorChecker and for all illumination configurations of Table 1 are also presented in Fig. 3 in bar chart format.

**Table 1:** Optimization results for simulations with free-running wavelengths.

$N_{\lambda }$	$\left\langle \Delta E_{2000}\right\rangle$	${\left\langle \Delta E_{2000}\right\rangle }_{\text{lip}}$	Wavelengths (nm)	Relative intensities (A.U.)
3	2.47	2.38	466, 537, 607	1, 1.07, 1.13
4	0.96	1.45	460, 518, 570, 619	1, 0.88, 0.92, 1.04
5	0.48	0.90	451, 497, 545, 589, 634	1, 1.12, 1.05, 0.93, 1.24

**Fig. 4:**
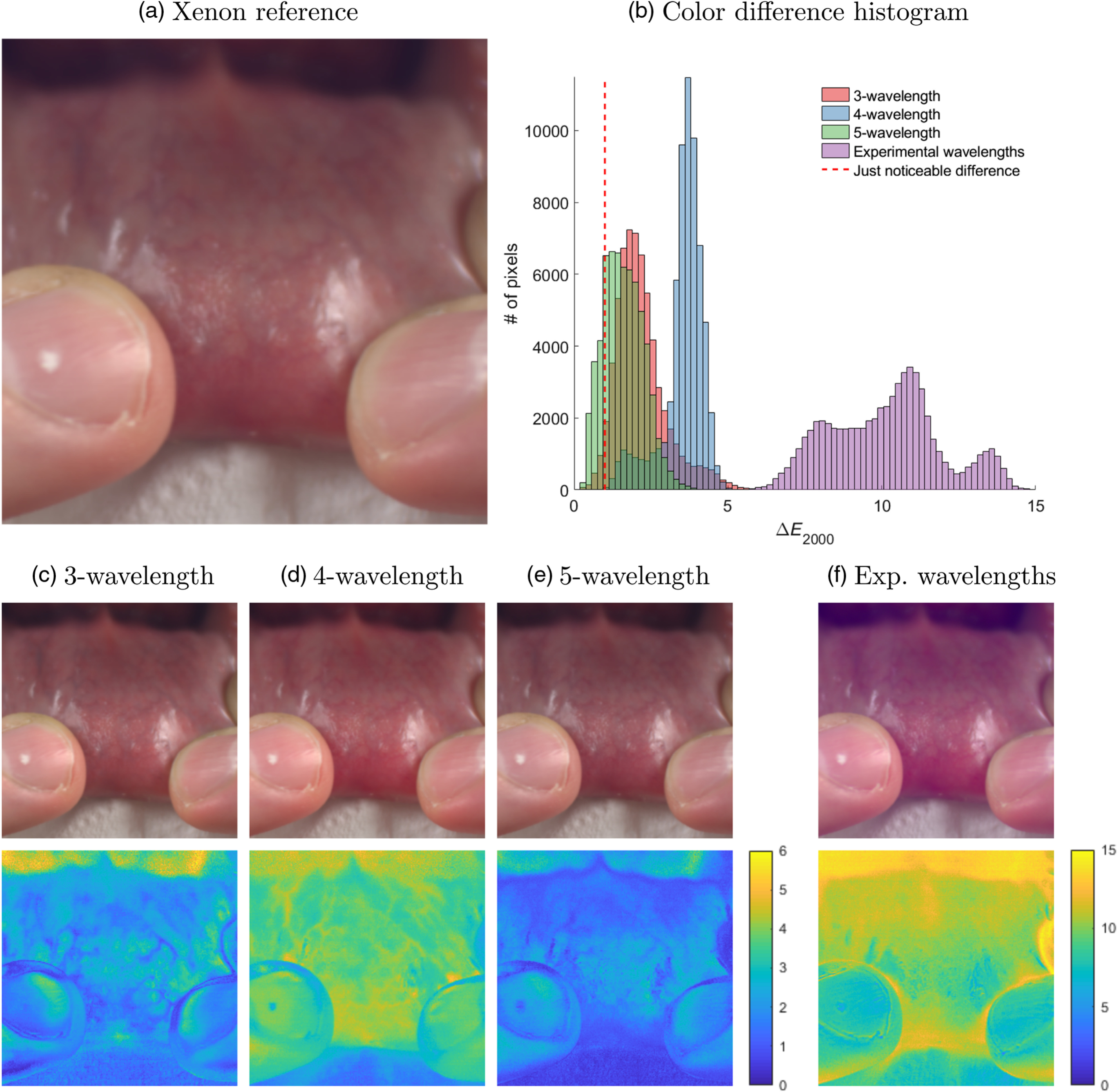
Reconstruction of true-color images of a lip from hyperspectral data. (a) Reference image under xenon illumination. (b) Color difference histograms of the $\Delta E_{2000}$ images [bottom images of subfigures (c)–(f)]. (c)–(f) Reconstructed color images (top) using the wavelength/intensity configurations listed in Table 2 and the $\Delta E_{2000}$ image (bottom) when compared to the reference image.

The optimization protocol was also performed with fixed wavelength values (see Table 2). All relative intensities are normalized to that of the first wavelength. The fixed wavelength values were selected from a list of commercially available lasers compiled from the catalogs of several suppliers (Oxxius, Coherent, Lasos, LaserGlow, and Cobolt). The wavelengths that were the closest to those identified in the free-running simulations were used in the intensity-only simulations. The last row of Table 2 describes the optimization results using the wavelengths from the multilaser source used in our experimental setup. Figure 4 consists of the images reconstructed from the lip reflectance hypercube, using the different illumination configurations presented in Table 2.

**Table 2:** Optimization results for intensity-only simulations. The first three rows used commercially available wavelengths closest to those presented in Table 1. The last row corresponds to the wavelengths from the 4λ laser source used in our experimental setup.

$N_{\lambda }$	$\left\langle \Delta E_{2000}\right\rangle$	${\left\langle \Delta E_{2000}\right\rangle }_{\text{lip}}$	Wavelengths (nm)	Relative intensities (A.U.)
3	2.60	2.18	465, 540, 607	1, 1.09, 1.11
4	2.49	3.55	460, 520, 561, 630	1, 0.65, 1.02, 1.50
5	0.86	1.61	454, 491, 546, 589, 633	1, 1.26, 1.26, 0.89, 1.39
4	3.61	10.50	405, 488, 561, 638	1, 0.11, 0.12, 0.15

### 4.2 System Characterization

**Fig. 5:**
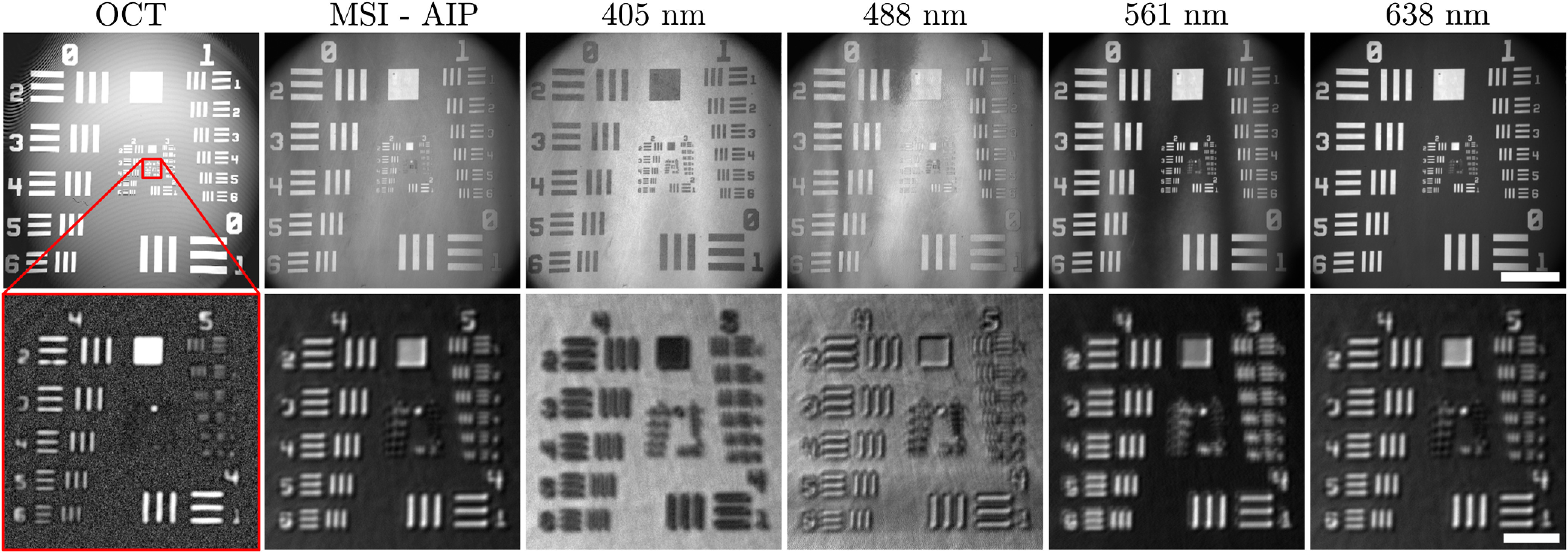
Multimodal imaging of a USAF resolution target. The top row is an $18\times 18\;{\mathrm{mm}}^{2}$ FOV, and the scale bar is 4 mm. The bottom row is a zoomed-in, $1.2\times 1.2\;{\mathrm{mm}}^{2}$ FOV, outlined in red in the top left image. The scale bar in the bottom images is 250  μm. The first column is OCT imaging, the second is an average intensity projection (AIP) with all four MSI wavelengths, and the last four are the individual MSI wavelengths extracted from a single acquisition.

We imaged a USAF 1951 resolution target to assess the lateral resolution of both modalities. We measured a lateral resolution of 28  μm for OCT and 22  μm for the overall MSI signal (total intensity projection). The individual wavelengths were found to have lateral resolutions of 23, 19, and 19  μm for 405, 561, and 638 nm, respectively. The resolution for 488 nm could not be measured due to the lack of contrast. The theoretical values, as defined for Gaussian beams using the Rayleigh criterion, are 27  μm for OCT and 9, 11, 12, and 14  μm for 405, 488, 561, and 638 nm, respectively (using NA=0.027). Images of the test target are presented in Fig. 5, for both large and small FOVs. The low contrast in the 488-nm images and the contrast reversal in the 405-nm images are caused by the low reflectivity/higher absorption of the chrome patterns at those wavelengths. All images for a given FOV were acquired concurrently.

### 4.3 Experimental Reflectance Measurements

**Fig. 6:**
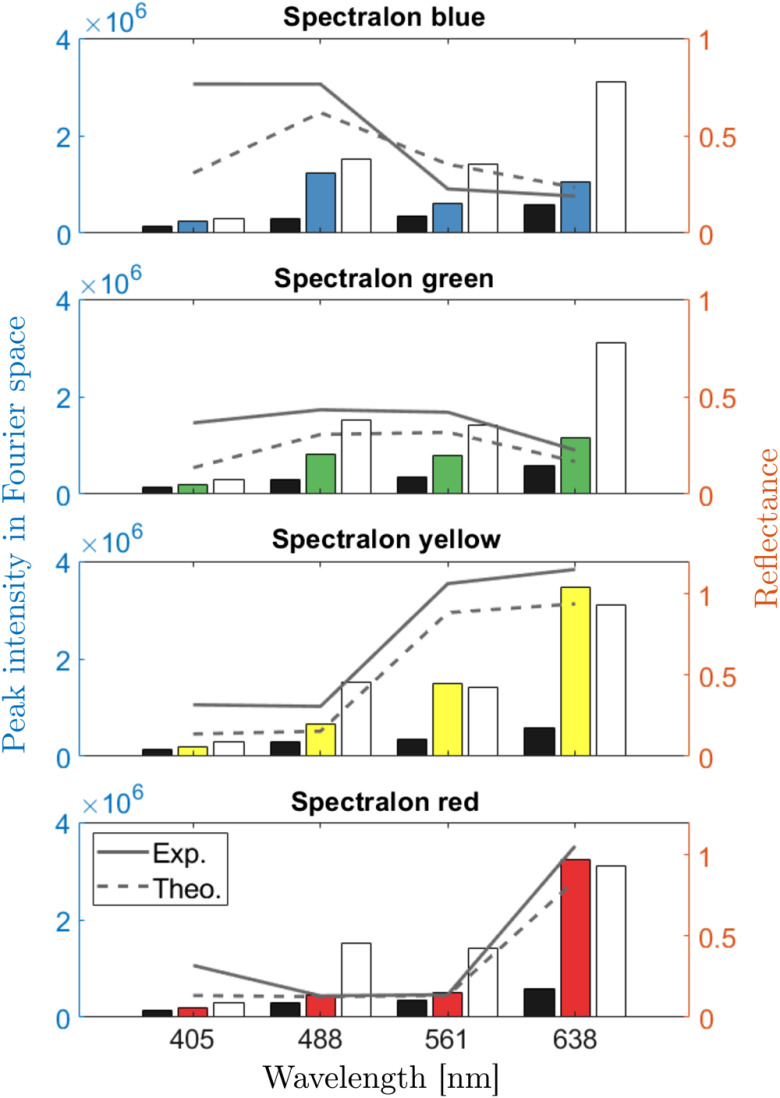
Experimental reflectance measurements on Spectralon color targets. (left axis) The bar plots depict the height of the peak in the frequency domain corresponding to each wavelength. For each wavelength, the dark (black bar), sample (colored bar), and reference (white bar) are shown. (right axis) The gray lines show the theoretical (dashed) an the experimental (solid) reflectance values, obtained from Eq. (9).

**Fig. 7:**
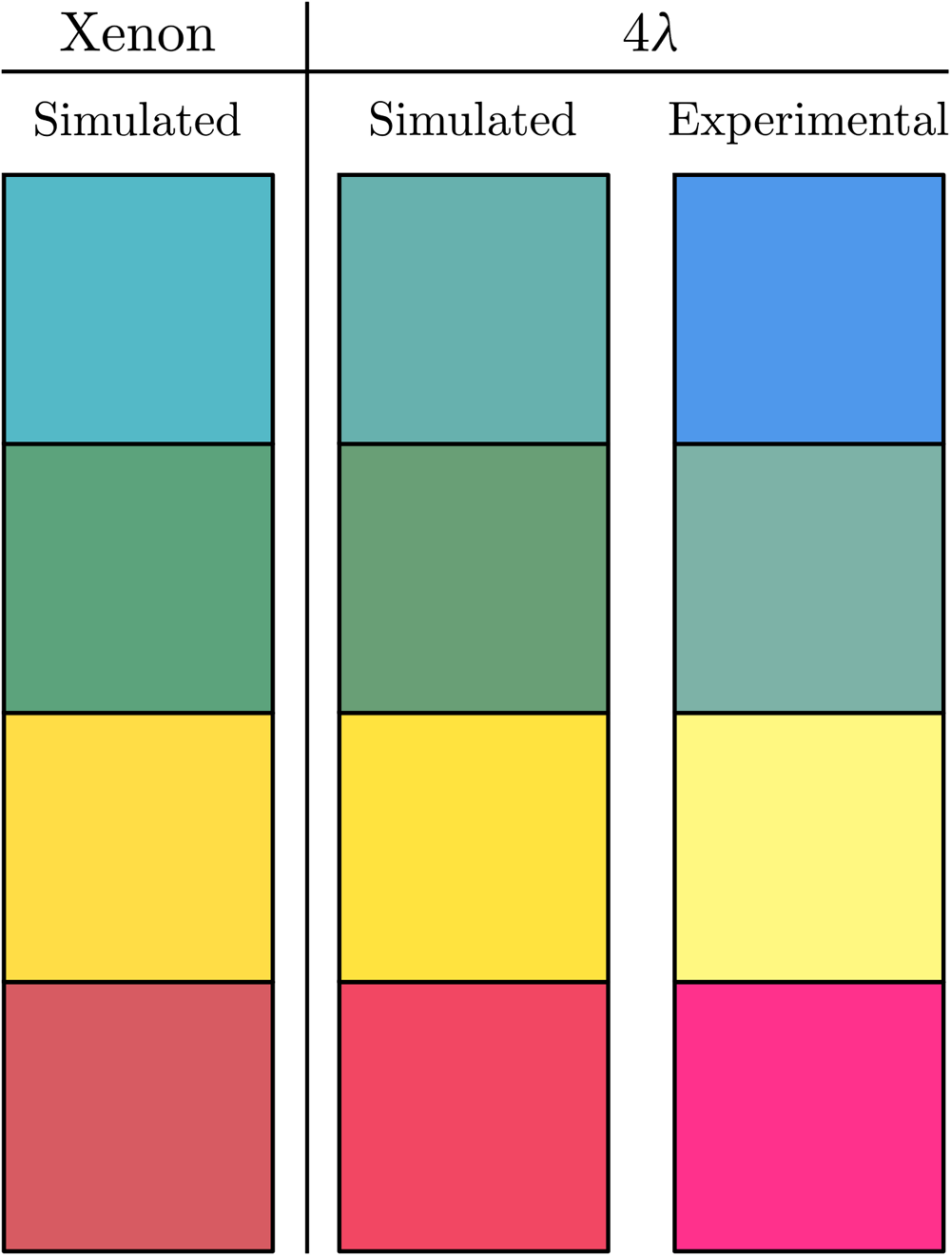
Reconstructed color of Spectralon color targets under three illumination schemes. From left to right: xenon illumination, theoretical color under 4λ illumination (using experimental wavelengths, see last row of Table 2), and experimental color under the same 4λ illumination. From top to bottom: blue, green, yellow, and red Spectralon targets.

To test color reconstruction with MSI, reflectance measurements were carried out on blue, green, yellow, and red Spectralon color standards (CSS-04-020 B/G/Y/R, Labsphere, USA), for which calibrated reflectance spectra are available. The reflectances at the four experimental wavelengths were defined by comparing sample, reference, and dark measurements as described in Eq. (9). The sample, reference, and dark measurements are presented in bar plot format in Fig. 6, where the black bar is the dark, the colored is the sample, and the white bar is the reference. Each subplot presents the intensity measurements at the four wavelengths for one color target. The bar plots and left axis depict the height of the peak in the Fourier domain while the gray lines and the right axis show the experimental and theoretical reflectance values. These reflectance values were then used to reconstruct the color of each color target, as illustrated in Fig. 7. The three columns show the color under xenon illumination, the theoretical color under our 4λ illumination, and the experimental color under the 4λ illumination.

### 4.4 Combined Imaging

**Fig. 8:**
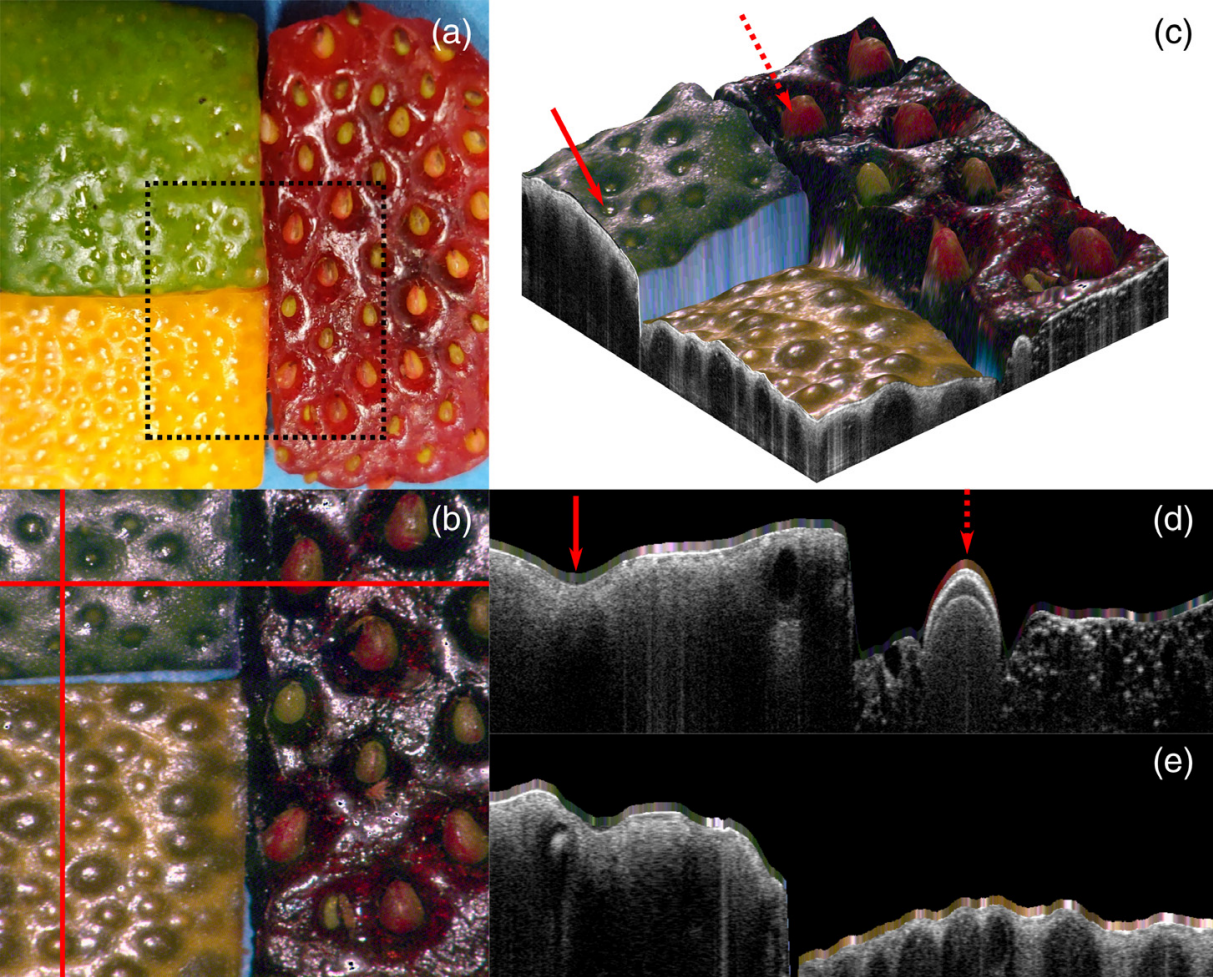
Combined OCT and MSI imaging of a fruit sample including mandarin peel, lime peel, and strawberry. (a) CCD picture of the sample. (b) True color image reconstructed from the MSI reflectance data. The red lines indicate the locations of the cross-sections presented in subfigures (d) and (e). (c) 3-D rendering of the fruit sample with color pixels super-imposed onto the sample surface. The red arrows identify features also visible in subfigure (d). (d), (e) OCT cross-sections with color pixels superimposed onto the sample’s surface.

Coregistered OCT and MSI imaging was demonstrated on biological samples exhibiting a broad range of colors, namely: a mandarine peel, a lime peel, and a strawberry, all placed on a blue piece of paper. The FOV was $7\times 7\;{\mathrm{mm}}^{2}$ and is depicted in Fig. 8(a) in dashed lines. OCT and MSI signal were acquired simultaneously. The OCT volume was processed for surface detection using a home-built algorithm. As both modalities are intrinsically coregistered and equally sampled in the lateral dimensions, each pixel in the reconstructed color image Fig. 8(b) corresponds to a single A-line in the OCT volume. It is, therefore, possible to apply one color pixel from the reconstructed color image to the surface of each A-line. This can be visualized in two dimensions (2D) [see Figs. 8(d) and 8(e)] or in 3-D [see Fig. 8(c)]. The reflectance values used to compute the color were obtained relative to a measurement of the white Spectralon standard. The blue vertical surface visible between the lime and mandarine peel in Fig. 8(c) is the blue paper visible in Figs. 8(a) and 8(b).

## 5 Discussion

### 5.1 Numerical Optimization for Color Reconstruction

The effectiveness of color reconstruction using multispectral illumination can be assessed qualitatively by considering Fig. 2, which shows that 4λ illumination enables near perfect color reconstruction. The color rendering capabilities of multilaser illumination can also be evaluated quantitatively using the $\Delta E_{2000}$ and the JND. As shown in Fig. 3 and described in the $\left\langle \Delta E_{2000}\right\rangle$ column of Table 1, the 4λ illumination achieves a mean color difference just below the JND threshold, whereas 5λ illumination drops well below it.

In Table 1, we demonstrate that it is possible to accurately reproduce the color of a sample under a reference illumination, using only a few laser lines. The results of the optimization process with free-running wavelength values are, however, difficult to reproduce experimentally as lasers are not available at all arbitrary wavelengths. Table 2 presents the result of the optimization process when the wavelengths are set to commercially available values, and only their relative intensities are optimized. When comparing the optimization results ($\left\langle \Delta E_{2000}\right\rangle$ column) of Table 1 with the first three rows of Table 2, we see that the accuracy of the color reconstruction with commercially available wavelengths decreases slightly but remains viable. The feasibility of accurate color reconstruction is further supported by Fig. 4, in which images of a human lip are reconstructed from hyperspectral data, using the wavelength configurations described in Table 2. A first observation, when comparing Figs. 4(c)–4(e) with the reference image [Fig. 4(a)], is that all images perform qualitatively well. In the case of Fig. 4(f), where the wavelengths deviate significantly from the optimal values outlined in Table 1, the color distortions are much more apparent. Typically, changing a wavelength by a couple of nanometers does not substantially degrade the color reconstruction. More significant deviations from the optimal wavelengths cause larger color differences, as may be observed in Figs. 4(d) and 4(f). In the context of this work, there is a significant mismatch between our experimental wavelengths and the optimal ones, as the multilaser source came preconfigured.

Another interesting observation is that, for intensity-only optimization with commercial wavelengths, 3λ outperforms 4λ for the lip images. However, it is important to note that 4λ still performs better than 3λ for the ColorChecker ($\left\langle \Delta E_{2000}\right\rangle$ column). As such, 4λ performs better for general color reconstruction (ColorChecker) but poorer for this particular biological sample (lip). This can be explained by the fact that, for 4λ illumination, the optimization protocol leads to slightly poorer color rendering for red colors, which happen to be dominant in this particular scene. The color reconstruction was also tested over multiple other hyperspectral datasets (not shown, data available online^35^) and, on average, 4λ performed better than 3λ. The slightly decreased performance of the 4λ configuration could also be explained by the fact that the commercial wavelengths depart more significantly from the ideal values presented in Table 1 than for 3 or 5λ illumination. It is interesting to note that, in the context of *in vivo* use, where certain colors are predominant, better color reproduction can be achieved by weighting the optimization process in favor of these dominant colors. However, care should be taken to not overemphasize the expected colors, as this may lead to a poorer reconstruction of the less frequent colors when they do appear in a scene, as might be the case when imaging pathological tissue (e.g., necrotic, inflammed, or infected tissue).

In light of these results, it is feasible to achieve high fidelity color reconstruction with 3, 4, or 5 commercial laser sources. A larger number of wavelengths is preferable as it leads to increased color rendering performances across a broad range of colors, as well as a reduced sensitivity to the wavelength selection. The latter has important practical implications as lasers at more exotic wavelengths may come at a significantly higher cost. Finally, the use of more wavelengths is also interesting as it may enable additional spectroscopic analyses.

### 5.2 System Performance

Our system was able to achieve concurrent and coregistered OCT and multispectral imaging (MSI), as shown in both Figs. 5 and 8. Figure 5 shows that it is possible to separate the different frequency components in the MSI signal to reconstruct images for each spectral band. However, the lateral resolution values for MSI were worsened compared to the theoretical values by factors ranging from 1.3 to 2.5. Two principal factors contribute to this effect. The first is the significant axial chromatic aberrations induced by the imaging lens (L in Fig. 1). Indeed, the focal shift between the MSI and OCT wavelengths is expected to be on the order of 1 mm. To not compromise the structural imaging with OCT, the system was optimized such that OCT was in focus while the MSI was slightly out of focus. This led to a broader point spread function (PSF) and thus poorer imaging resolution. These chromatic effects may be corrected through the use of more advanced color-correcting optical components or by replacing any refractive elements with reflective optics. The use of reflective optics is also interesting because it removes any problems associated with backreflections, which contribute to background noise. In our experiments, backreflections were apparent in the OCT images when the scanning beam had a normal incidence to the imaging lens leading to a localized decrease in SNR. The second effect is the multimodal nature of the VIS illumination. Indeed, the MSI illumination is carried out through the fiber core of the DCF, which has optical properties identical to an SMF28 fiber. All four MSI wavelengths are well below the cut-off wavelength for such a fiber (1250 nm), and the propagation in the DCF core is therefore multimodal. This causes distortions in the wavefront, which lead to an aberrated PSF and poorer lateral resolution. This effect could be addressed through the use of photonic crystal fibers, which are single mode at all wavelengths. However, this is not feasible in this setup as the DCFC technology is currently not compatible with such fibers. While these effects result in suboptimal optical performance, the system’s performance is adequate for practical intents and purposes. Indeed, if diffraction-limited resolution was achieved for the VIS wavelengths, a much denser sampling would be necessary to satisfy the Nyquist criterion. This would lead to much longer acquisition times and a significant oversampling for OCT.

It is also relevant to notice that through the combined use of the DCFC and the WDM, the system is entirely fiber-based. As such, it is highly robust to movement and vibrations and requires simplified laser safety considerations. These factors may facilitate future implementation in a clinical setting.

### 5.3 Reflectance Measurements

The reflectance measurements shown in Fig. 6 indicate significant differences between the theoretical and the experimental reflectance values for all four Spectralon samples. This is due to the difference in measurement geometry. The collected intensity for any given wavelength and imaging geometry can be described as a Beer–Lambert law that accounts for the different path lengths inside the sample, assuming a homogeneous absorption coefficient. This expression can also be reformulated as the expected value of the attenuation for a given path length distribution

$$I(\lambda )=I_{0}(\lambda )\int_{0}^{\infty }e^{-\mu_{a}(\lambda )l}\rho (l)dl=I_{0}(\lambda )\langle e^{-\mu_{a}(\lambda )l}\rangle ,$$ (10)

where $I_{0}(\lambda )$ is the initial intensity of the collected light without attenuation, $\mu_{a}(\lambda )$ is the absorption coefficient, l is the photon path length, and ρ(l) is the photon path length distribution (PPLD), which is defined by the sample scattering properties and the illumination/collection geometry. Any reflectance measurement is then defined as the ratio of the measured intensity with and without attenuation but with the same PPLD: $R(\lambda )=I(\lambda )/I_{0}(\lambda )=\langle e^{-\mu_{a}(\lambda )l}\rangle$. Two reflectance measurements of the same sample (fixed $\mu_{a}$) could then only be expected to return the same value if they also share the same PPLD. The theoretical reflectance values described in the specification of the Spectralon color standards are determined by illuminating the sample with a large diameter pencil beam and collecting all of the diffuse light with an integrating sphere. In the experiments presented in this work, both illumination and collection are carried out through a single fiber and the imaging optics (i.e., in a confocal geometry). As such, the collected photons have a significantly different propagation regime than in the widefield case. It can, therefore, be expected that the PPLD and the measured reflectance values will differ from the theoretical values.

It should be noted that some of the measured reflectances return values greater than 1 (see Fig. 6 for the yellow and red Spectralon targets). This effect may be attributed to the measurement uncertainty associated with the sample, reference, and dark measurements. The resulting relative uncertainty on the measured reflectance was on the order of 15%. All measured reflectances over unity were still within the range of 1±0.15. It is also possible that the scattering properties of the white and colored Spectralon targets are not identical due to the presence of the additional chromophores. The PPLD would then be different, which may result in reflectance values greater than one.

The variation in measured reflectance caused by the imaging geometry also influences the reconstructed color, as shown in the third column of Fig. 7. It is, therefore, inevitable that color differences appear between acquisitions with video cameras and widefield illumination, and point scanning systems such as the one presented in this work. These differences may be accounted for by defining approximate correction factors, which would consist of both system and sample specific components. Such correction factors require the study of the propagation of light in the sample as a function of the sample’s optical properties and the system-specific imaging geometry. The theoretical framework for this analysis has already been developed in a nonimaging technique called single fiber reflectance spectroscopy (SFR) where reflectance measurements are carried out with single-fiber probes in direct contact with the sample.^36^^–^^39^ These SFR models may be adapted for noncontact imaging with the DCFC setup to accurately determine the correction factors necessary to recover the reflectance as measured by a video camera. Furthermore, these models may be used to estimate both the optical and physiological properties of biological samples.

### 5.4 Multimodality, Spectroscopic Analyses, and Future Work

The clinical utility of combined OCT and MSI lies in the simultaneous access to both depth-resolved structural information and surface color. OCT may reveal subsurface structural pathological features, and color information provides a familiar perspective that may also be used for diagnostic purposes. Figure 8 shows such combined imaging. It demonstrates the feasibility of reconstructing high-resolution color images coregistered with OCT imaging. Furthermore, MSI is not limited to color reconstruction. Indeed, it would be relatively simple to change or add wavelengths that would allow other types of clinically valuable spectroscopic analyses. Such analyses may include oximetry or blood hematocrit measurements that may be indicative of abnormal/pathological conditions such as inflammation or neoplastic angiogenesis. Furthermore, the same optimization process presented here could be used to reproduce sample appearance under narrowband imaging illumination. The addition of wavelengths would not compromise color reconstruction as the signal from each wavelength can be addressed independently. The spectroscopic information could then be processed and overlayed onto the OCT data for simultaneous visualization. Such analyses would rely on accurate propagation models to infer optical properties from the measured reflectance values. Future efforts will, therefore, be concentrated on adapting the afore-mentioned SFR models to our imaging setup.

## 6 Conclusion

We have presented several key steps in the development of a combined OCT and MSI system. This includes an optimization procedure to guide the selection process of MSI wavelengths and their relative intensities for optimal color reconstruction. Using this procedure, we demonstrate that it is theoretically possible to reproduce the color of a sample as it would appear under broadband illumination using only a few wavelengths. Furthermore, we developed a fiber-based system that allows concurrent and coregistered imaging with both modalities. MSI signals are multiplexed in the frequency domain, acquired using a single detector and separated in postprocessing using Fourier analysis. We demonstrate the system’s ability to carry out high-speed, high-resolution, and simultaneous imaging with both modalities. Future efforts will be centered around modeling of light transport in samples to extract optical properties as well as better reconstruction of color.
